# Ethnomedicinal Uses of Plant Resources in the Machhapuchchhre Rural Municipality of Kaski District, Nepal

**DOI:** 10.3390/medicines6020069

**Published:** 2019-06-23

**Authors:** Mahendra Adhikari, Rashmi Thapa, Ripu Mardhan Kunwar, Hari Prasad Devkota, Prakash Poudel

**Affiliations:** 1 Department of Pharmacy, Novel Academy, Purbanchal University, Pokhara 33700, Nepal; mahendraadhikari2052@gmail.com; 2 School of Health and Allied Sciences, Pokhara University, Pokhara 33700, Nepal; rasmithp@gmail.com; 3 Department of Geosciences, Florida Atlantic University, 777 Glades Road, Boca Raton, FL 33431, USA; ripukunwar@gmail.com; 4 Graduate School of Pharmaceutical Sciences, Kumamoto University, 5-1 Oe-honmachi, Chuo ku, Kumamoto 862-0973, Japan; devkotah@kumamoto-u.ac.jp

**Keywords:** ethnomedicine, Kaski district, disease and ailments, medicinal plants, Nepal

## Abstract

**Background:** Medicinal plants are being used by the majority of the population throughout the world for their primary health care needs. The reliance is also prevalent in Nepal, aided by its ethnic and biological diversity. This study aimed to catalogue the ethnomedicinal knowledge of plants used by local people of Machhapuchchhre Rural Municipality of Kaski district. **Methods:** Data were collected between February 2017 and April 2018 from eight different villages of the Kaski district by using semi-structured interviews, guided field works, focus group discussions, and in-depth interviews. The reported traditional uses were supported by local assistants, cataloguing vernacular names and crosschecking with the earlier published and gray literature. **Results:** A total of 105 medicinal plants, belonging to 58 families and 99 genera were documented to treat 70 different diseases and ailments. The highest numbers of plants (37) were used for gastrointestinal disorders and the lowest (4) were used for female genital disorders. Commonly used parts were underground portions (28 species) followed by fruits and seeds (25 species each). The most preferred dosage form was juice, used for 50 ailments, and the oral route was the most favored route of administration (77 species). The medicinal properties of 22 plant species were found hitherto unreported in the district. **Conclusions:** The study area was found to be rich in plant resources and the people have ample knowledge on the use of medicinal plants. Due to a lack of proper documentation, conservation, and cultivation practices, valuable plant species are at risk of extinction. Thus, appropriate conservation measures and scientific assessment of plant-lore in the district is immediately required.

## 1. Introduction

Traditional medicine has played a crucial role in healthcare system for a long time [1]. The World Health Organization (WHO) defines traditional medicine as the “Sum total of the knowledge, skills, and practices based on the theories, beliefs, and experiences indigenous to different cultures, whether explicable or not, used in the maintenance of health as well as in the prevention, diagnosis, improvement or treatment or physical and mental illness” [2]. Thus, traditional medicine is strongly bonded to nature and dependent on natural resources and culture [3,4]. Historically, plant and animal derived products were the only source of nearly all medicinal preparations [5]. Among them, medicinal plants have been the major source of crude drugs used in different traditional medicine systems throughout the world [6,7,8]. The discovery of the medicinal properties of plants came from long self-experiments, which took centuries for establishment [9]. Due to the lack of appropriate strategies for screening, the use of plant-derived products has diminished in the past two decades [5]. It is estimated that only 10% of the world’s biodiversity has been assessed for their medicinal properties [10]. However, the utilization and determination of their therapeutic potential have not been prioritized to foster the quality of livelihood [11]. In recent years, the application of emerging technology advances in biological activity screening and chemical analysis have increased the interest of researchers towards naturally derived compounds. Lots of active constituents are to be investigated, from both terrestrial and marine sources [5]. In this scenario, the extensive survey of traditionally used plants in their natural habitats will definitely contribute to the identification of medicinally important plants.

In Nepal, it is estimated that only 17% of people live in urban areas and have access to modern medicine and the rest of the population still depend on the traditional system of medicine for their basic health care needs [12,13,14,15,16]. Traditional healers and elderly people learned folklore through apprenticeships to treat common health disorders based on their ethnomedicinal knowledge [11,17]. However, such knowledge still remains unexplored scientifically [11,13]. Medicinal plant resources have been equally used in the traditional scholarly medical systems, including Ayurveda, Tibetan and Unani, and in folk medicine [18]. Nepal occupies only 0.9% of the world’s land area, but possesses nearly 7% of the total medicinal herbs of the world [19,20]. Over 2000 plant species have been used frequently in rural, remote, and suburban areas of Nepal [21,22]. The uses are associated with the 125 diverse ethnic groups of the country [23]. Some ethnic societies consist of their own healing systems [7,11] and knowledge is transferred orally through generations [7]. However, the local healing system is affected due to changes in lifestyles as a result of globalization, increasing population, land-use change, and global warming. The collection and use of plants substantial for local livelihood, primary health care, and pharmacology [24] has now been severely threatened due to local people’s changing perceptions and their context-specific socioeconomic and cultural transformations [25]. Thus, traditional medicine in rural and remote areas of Nepal has experienced a substantial change in the recent decades [26] and it has declined in the absence of proper documentation.

There are a handful of ethnomedicinal studies carried out in Kaski district [27,28,29,30,31], while none is from the rural and remote villages of the Machhapuchchhre rural municipality. The present study, therefore, aims to investigate and document the plant-based indigenous knowledge of local people and explore their traditional uses. We hypothesized that the rural and remote areas of Nepal are rich in medicinal plants and the inhabitants possess unique knowledge of plant use. We also hypothesized that the richness of plants and the knowledge heritage of the area are positively associated. 

## 2. Materials and Methods 

### 2.1. Study Area

Kaski district lies between 83°40’ east to 84°12’ east longitude and 28°06’ north to 28°36’ north latitude and between 450 m and 8091 m elevation above sea level. It comprises a diverse topography (hills, midhills, mountains, Himalaya) and the following five common bio-climates: Sub-tropical, temperate, temperate cold, alpine, and tundra. The average rainfall is about 2500 mm per annum and the maximum temperature is 34 °C during the summer (April–July). It is estimated that 46.4% of the total area of the district is occupied by four types of forest, including subtropical broadleaf forest, temperate forest, subalpine forest, and alpine forest [32].

### 2.2. Sampling

**Figure 1:**
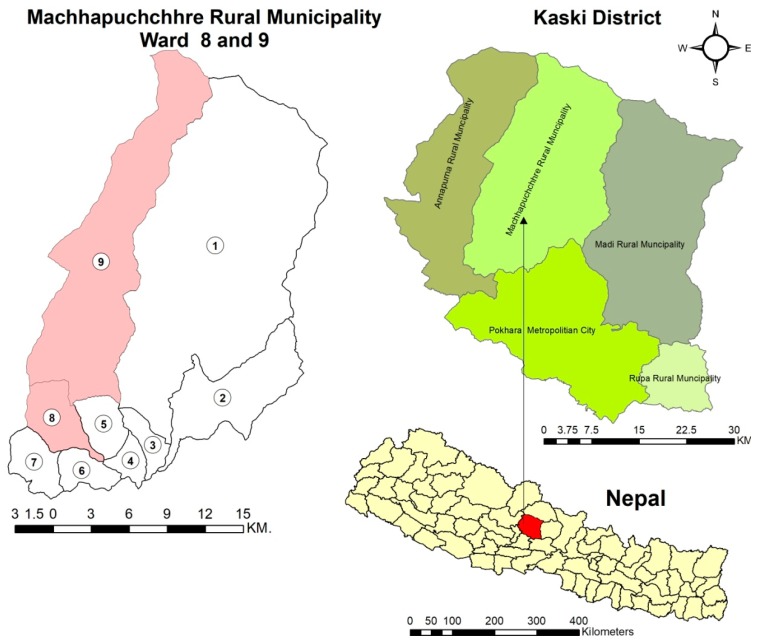
Map of the study area (Machhapuchchhre Rural Municipality) in the Kaski district of Nepal.

The present study was conducted in purposively selected eight remote and rural villages (Koleli, Lwang, Ghalel, Lumre, Sakhu, Siu, Sidhing, and Idikhola) of the Machhapuchchhre rural municipality of the Kaski district (Figure 1). The study villages lie in the Annapurna Conservation Area (ACA), the largest protected area of Nepal [33]. Being located inside the conservation area, the collection of medicinal plants is restricted. The villages are populated with 21,868 inhabitants from 5512 households. The major ethnic groups are Brahmin, Gurung, Kami, Magar, and Chhettri. The communicable language is Nepali but the local people prefer to speak their own dialect, like Gurung and Tamang [32]. Rice, wheat, maize, millet, mustard, potato, etc. are the major crops, while, buffalo, goat, chicken, and cow are the major livestock. Economically, people mostly rely on agriculture and occasional trading of grains, livestock, and cash crops like tea, broom grass, cardamom, Himalayan bamboo shoot, fiddle head fern, and medicinal plants [34]. Although both modern and traditional system of medicines are practiced in this area, there is very limited access to modern allopathic medicine having only one primary health center and seven health posts [32]. Therefore, local people rely on the persistent traditional system of medicine for their basic health care needs.

### 2.3. Data Collection and Analysis

The year-long fieldwork was carried between February 2017 and April 2018. A total of 6 field visits were made in each study site. The four main data collection strategies such as Semi-structured interviews, guided field surveys, focus group discussions, and in-depth interviews were used following Martin, 1995 [35]. Ethical approval and prior oral consent was obtained from the Institutional Review Committee (IRC), Pokhara University, Nepal, ACA office and the study participants. During the study, a total of 49 traditional healers including *Dhami* and *Jhankri* (11), *Pujari* (4), *Vaidhya* (3), and a local herbalist (27) were reached out for study and only 45 allowed us to interview further. Four traditional healers refused to respond to our work because they lamented that the methods of treatment shared among others is disgraceful. A semi-structured questionnaire was designed for the interviews. Among the respondents, 85% were male and 89% were above 50 years of age and 72% were literate. Concerning the ethnicity of participants, Brahmins were found to be dominant (53 %), followed by Gurung (16%), Lama and Tamang (18%), and Dalit (13%).

In situ interviews were taken at the homes of the participants. Questionnaires were explained in Nepali and local dialects. Questions used during the interview sought information including the socio-demographic description of the participants, the local names of plants, plant habitats, parts used, dosage forms, the route of crude drug administration, and disease and ailments treated by the plants. Before conducting extensive interviews, questionnaires were pre-tested with five informants and modified as required. Similarity, dissimilarity, and new indication of plants were confirmed by comparing with previous relevant data [6,7,11,12,13,22,27,28,30,31,36,37,38,39,40,41,42,43,44,45,46,47,48,49,50,51,52,53,54,55,56,57,58,59,60,61,62,63,64,65,66,67,68,69,70,71].

The ethnomedicinal uses of plants for various diseases and ailments were analyzed following the International Classification of Primary Care (ICPC) and compared with the literature. The comparison analysis helped to sort out the novelty of the findings. The data were entered in Microsoft Office Excel 2010 to analyze the information regarding plant families, habits, parts used, dosage forms, routes of administration, and the number of ailments treated. Data were expressed in terms of number and percentage. Voucher specimens collected during forest walks were identified using the literature, local vernacular names, and experts’ knowledge. Herbarium voucher specimens were cross-identified at the National Herbarium and Plant Laboratories (KATH), Godawari, Lalitpur, Nepal and housed in Novel Academy Herbarium, Novel Academy, Pokhara, Nepal. 

## 3. Results

### 3.1. Medicinal Plant Diversity and Uses

Altogether, 105 medicinal plants belonging to 56 families and 99 genera were recorded. Out of 56 families, Asteraceae and Poaceae were the most dominant families, each with 9 species, followed by Fabaceae and Solanaceae (7 species each), Lamiaceae (5 species), Rutaceae (4 species), Amaranthaceae, Apiaceae, and Rosaceae (each with 3 species). The remaining 47 families possessed less than 3 plant species each. The scientific name, family name, local name, and folk uses of the plants are summarized in Table 1.

### 3.2. Use of Plants for Primary Health Care

It was found that local people used 105 medicinal plants to treat 70 different types of diseases and ailments (Table 1). These diseases and ailments were grouped into 13 different categories (Table 2) based on the International Classification of Primary Care (ICPC) i.e., digestive (37 species), skin (27 species), respiratory (19 species), musculoskeletal (12 species), circulatory (11 species), endocrine, metabolic, and nutritional (10 species), pregnancy, childbearing, family planning (5 species), eye (4 species), urinary (4 species), neurological (3 species), ear (2 species), female genital system (1 species), and general and unspecified (35 species). The information regarding the use of plant species for specific categories of diseases and ailments is listed in Table 2. Similarly, the biomedical and emic use reports are presented in Table 3.

### 3.3. Plant Parts Used and Their Growth Forms

**Figure 2:**
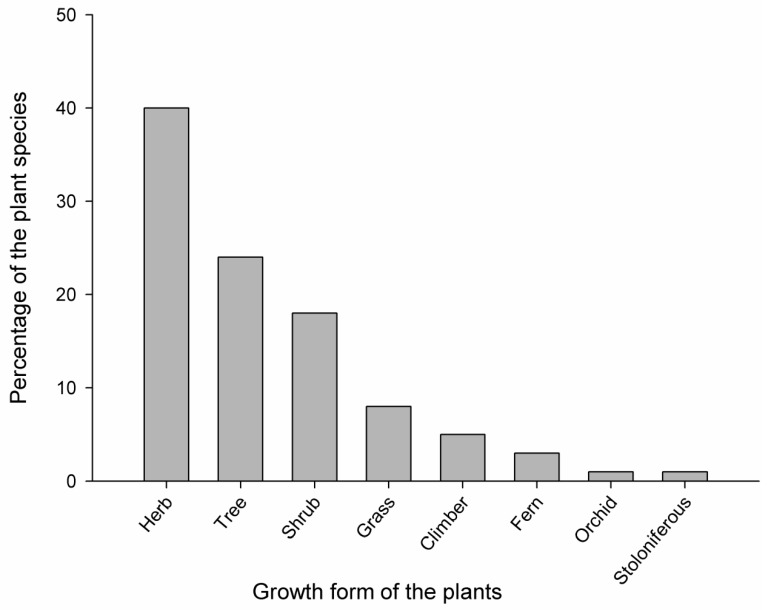
Growth form of the plant species used by the local people.

Almost all parts of the plants were used to prepare medicine (leaf, root, rhizome, tuber, bulb, pseudobulb, corm, stem, bark, pith, flower, fruit, seed, bud, shoot, and whole plant) however, the most common plant parts used were underground parts including the root, rhizome, tuber, bulb, pseudobulb, and corm (used in 28 species), followed by fruit and seed (25 species), leaf (23 species), stem, bark, and pith (21 species), whole plant (17 species), bud and shoot (8 species), and flower (4 species). Additionally, the data of growth forms of plants indicate that most of the people used herbs (40%), followed by trees (24%), shrubs (18%), grasses (8%), climbers (5%), ferns (3%), orchids (1%), and stoloniferous (1%). Information regarding the number of plant species of each growth form is demonstrated in Figure 2.

### 3.4. Dosage Forms and Routes of Administration

**Figure 3:**
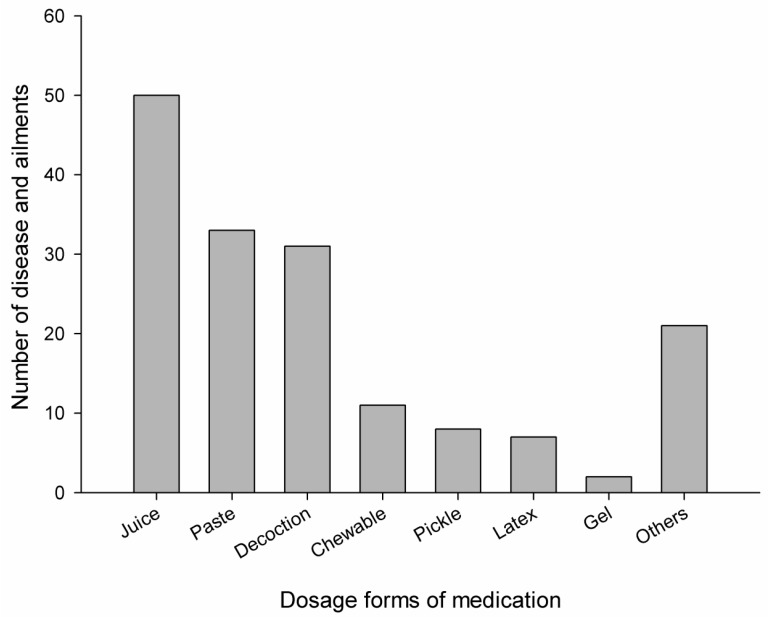
Dosage forms of medication in the treatment of different diseases and ailments by the local people.

**Figure 4:**
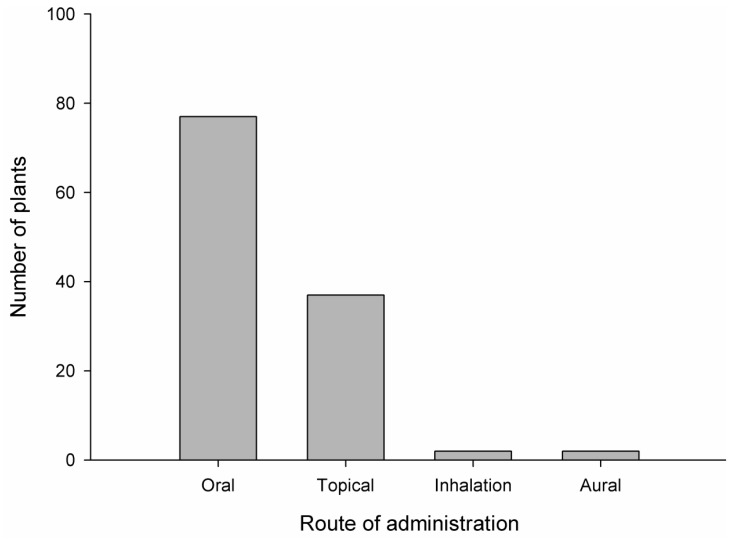
Routes of administration and the number of plants used by the local people.

The study revealed that medicinal plants were mostly used in the decoction, juice, powder, paste, gel, latex, and pickled forms. Less frequently used formulations were roasted, vapor, boiled, and in traditional dishes like *Puwa* (plant powder cooked with rice flour in butter). Few plant species were also taken in chewable form. It was found that the most frequently used dosage form was juice (50 ailments), followed by paste (33 ailments), decoction (31 ailments), chewable (11 ailments), pickled (8 ailments), latex (7 ailments), gel (2 ailments), and other (17 ailments). Similarly, the oral route was found to be the most preferable route (77 plants) followed by the topical route (37 plants), the inhalation route (2 plants), and the aural route (2 plants). None of the plant preparations were taken through the parenteral route. The diagrammatic representation of the dosage form and their routes of administration are displayed in Figure 3 and Figure 4, respectively. 

## 4. Discussion

### 4.1. Plants and Usefulness

Records of higher numbers of useful plant species from the plant families Poaceae, Asteraceae, and Fabaceae were already manifested in Nepal [82]. Poaceae, Asteraceae, and Fabaceae are the families with a large number of genera and species. It is often hypothesized that there exists a positive correlation between plant use/knowledge and plant density, diversity, and the habitat diversity [54,55]. If the area is biodiverse, the use of plants is heterogeneous and if the area is less diverse (abundant), the use of plants is homogenous [83]. The higher number of ethnomedicinal plants was found to be associated with the area rich in biodiversity. 

**Figure 5:**
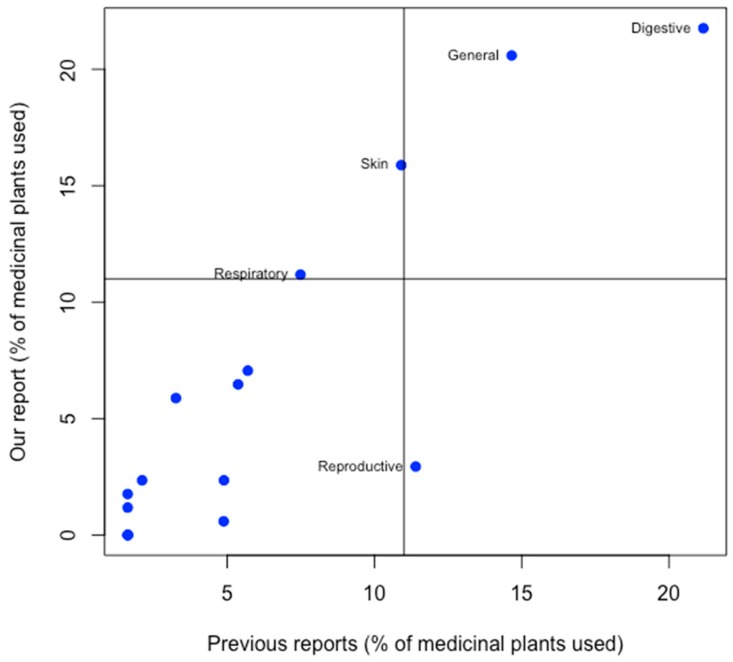
Comparison of the medicinal value of plants in the present study with previous studies in the Kaski district.

It was found that the people of the Kaski district are rich in ethnomedicinal knowledge and mostly rely on plant-based remedies for common health problems like gastrointestinal disorders, respiratory disorders, musculoskeletal disorders, cardiovascular disorders, etc. The number of plants used to treat gastrointestinal disorders was found to be the highest (Figure 5), which is convergent to other studies considered when reviewing this study [22,37,39,41,48,53]. Most of the gastrointestinal problems were due to irregular dietary habit, poor hygiene, and contaminated food. Respiratory, diarrheal, and infectious diseases are also prevalent in Nepal and the country’s sociological and topographical complexities burden health and sanitation [84]. Life in rural areas is further complicated [56] because of the effacement of traditional knowledge. Due to a lack of health education, people live with poor hygiene are vulnerable to many diseases. Benign health issues were frequently undermined because of a lack of health education. Some of the skin diseases like photo dermatitis [85], were caused due to prolonged exposure to sunlight during working hours. People suffered from fungal infections in the hands and feet, due to exposure to soil. Many people had musculoskeletal disorders due to greater physical stress, sprains, and fractures.

### 4.2. Perspective of People Towards Ethnomedicine

We found that the elderly people and traditional healers were rich in ethnomedicinal knowledge and were more interested to conserve plants than younger people. Villagers believed in traditional healers, such as *Dhami*, *Jhankri*, and *Pujari*, as God’s messengers and with super healing powers. They believe that plants become more medicinal when processed spiritually and materially. The traditional healers primarily used local medicinal plants to treat diseases. Besides local plants, they also used other important natural products available in other parts of the country. They generally exchanged their grains (such as rice, maize, etc.) with herbal ingredients brought by the transhumants from the Himalayan region.

Some of the elderly people claimed that they have not taken any allopathic medicine in their lifetime. However, they were less conscious of documentation and were unwilling to share their knowledge with others. They believed that if such secrets and their inherent knowledge is shared, the method does not work. They did not have any formal written documents about the use of medicinal plants. Most of the people in this area were not alarmed by the possible extinction of their indigenous knowledge. Few participants even told that traditional healers were remembered in their needs but not rewarded in return. Studies showed that younger people have little knowledge about plants and were inclined to allopathic medicines. However, they were interested in the documentation of their forefather’s knowledge. Existing ethnomedicinal knowledge of these indigenous inhabitants has only been transmitted through the verbal prescription. This inherent and practical based knowledge is going to collapse soon, due to the declining interest of youngsters towards the value of traditional medicine and lack of specific plans and policies from the local government. 

### 4.3. Plant Parts Used and Their Growth Form

Among the choice of plant parts, underground portions were found to be frequently used. Our finding bears a resemblance with much previous literature on the use of plant parts [22,40,43,45,46,48,50,51,57,58,59]. Regarding the preference of underground parts, the root was used in most of the cases, as it generally contains a greater amount of active principles, in comparison with other parts [60]. Like roots, leaves are also vulnerable parts of the plant and have a greater role in the plant defense system and possess higher concentrations of bioactive secondary metabolites [49,61,86]. This finding may be beneficial for choosing these parts for bioprospecting and bioactivity determination. However, improper and massive collections of roots may lead to complete destruction and decline of the plant from nature [62]. Herbs were found to be dominant growth form over others, being frequently used in Himalayan folk medicine and other countries [7,13,22,30,39,40,43,48,50,51,52,53,56,58,59,63,64,65,66,86]. The reason may be that our study site is located at a higher altitude, where trees are less abundant and other forms of plants are common [22]. It also may be due to the greater ease of collection, storage, transportation, and extraction of active compounds from herbs than other forms [51]. Herbs also contain higher amounts of secondary metabolites for their life strategies [67]. Herbs are relatively easy to cultivate, so they can easily fulfill the demand if needed in higher amounts [68]. Hence, our finding highlighted that the selection and use of herbs depend on factors like their phytochemical profile, their natural habitat, and their accessibility, partially supporting ecological apparency hypothesis.

### 4.4. Dosage Forms and Route of Administration

In this area, the juice was used most frequently, followed by paste, decoction, chewable, pickled, latex, and gel. This information resembles the data published by other studies carried out in Nepal and other countries [7,51,53,59]. The preference of juice may be due to the ease of preparation and its effectiveness [58]. It may also be due to the presence of a greater amount of active principles extracted in juice than other dosage forms [66]. It was also found that most of the herbal plants were used with hot water. Some plants were used along with milk, ghee, and honey. For example, flour of *O*. *sativa* and powder of *B*. *ciliata* were prepared in a ghee base, both for oral and local application, for backache and inflammation.

Most of the dosage forms were freshly prepared when needed. For example, a fresh paste of *G*. *hirta* leaves and latex of *M*. *pustulata* was applied around boils for faster healing purposes. A single plant can be used to treat many disorders. Examples of such plants included *A*. *bidentata*, *A*. *sativum*, *A*. *sessilis*, *B*. *ciliata*, *C*. *verutum, C*. *esculenta*, *D*. *diandra*, *J*. *curcas*, *L*. *cubeba*, *M*. *arboreus*, *M*. *jalapa*, *O*. *tenuiflorum*, *O*. *sativa*, *R*. *arboreum*, *R*. *ellipticus*, *R*. *nepalensis*, *S*. *melongena*, *T*. *latifolia*, *Z*. *armatum*, and *Z*. *officinale*. Plants were also used in combined forms. People said that paste and juice of *C*. *asiatica* and *C*. *dactylon* are used together for typhoid. Similarly, people confirmed that the paste of three plants, *A*. *sativum, Z*. *armatum*, and *Z*. *officinale*, is useful for treating snakebites by the expulsion of venom from the snakebite. The increased effect of the plants used together may be due to their synergistic effect. This increased bioactivity of the combined plants opens the door for the identification of compounds with similar mechanism of action. While comparing the choice of routes of drug administration, the oral route was found to be the most preferable route in this study area. The data from other studies also showed that people favor the oral route for medications [22,41,48,51,56,59,61,64].

### 4.5. Comparison of the Reported Uses

We found that there are 47 plant species that have similar uses to treat 42 different ailments. *A*. *bidentata* was used in typhoid, tonsillitis, and toothache in this area. However, the same plant was found to be useful in the treatment of urinary disorder and indigestion in the Kavrepalanchok district [7] and asthma in Macchegaun, Kathmandu [36]. Another plant, *A*. *calva*, was used for dermatological disorders in the Rupandehi district of western Nepal [69]. However, this plant is used for gastrointestinal disorders like an intestinal worm, gastritis, and flatulence in the Kaski district. The rhizome of *A*. *calamus* was used in respiratory disorders like cough, chest pain, and asthma in our study area, which was also practiced in different regions of Nepal, like Rasuwa [13], Rupandehi [44,53], Humla [22], Macchegaun [36], Makawanpur [40], Gulmi [63], Dolpa [46], Nawalparasi [49], and Ghandruk [31]. The bulb of *A*. *sativum* fried in mustard oil was applied to treat earaches in Pakistan [70]. Similarly, its oil and fresh bulbs are used to cure skin rashes and blood pressure in the Kavrepalanchok district [7]. The decoction of this plant is taken for gastritis, flatulence, diarrhea, and dysentery in our study area and the paste of bulb is applied topically for snake bites. We found that people used the juice of *A*. *vera* for burns and boils. However, the same plant is used for diabetes in the Panchase village of the Kaski district [28], fever and cough in the Makawanpur district of Nepal [40], menstruation cramps, intestinal worms, and constipation in the Badgaun village of the Gulmi district [63], and rheumatism, constipation, and backache in Pakistan [56]. The importance of this plant for burns and boils in our area coincides with the accounts reported in Panchase, Kaski [28], Machhegaun, Kathmandu [36], and the Rudraprayag district of neighboring country India [66].

The inhabitants of Dekhatbuli of Kanchanpur districts used a paste of *A*. *sessilis* to treat wounds, venereal diseases, menstrual disorder, fever, and bloody dysentery [11]. The same plant has been reported for scabies in Rupandehi, Nepal [69] and bleeding, cuts, and wounds in Machhapuchchhre, Kaski, and Gulmi, Nepal [63]. Few previous studies showed leaves of *B*. *pinnatum* are beneficial for wounds, boils, skin burn, and are also applied to remove pus from the ear and vagina in Rupandehi, Nepal [69] and the plant is used to relieve ear pain in the Kaski district. Local people have been using the apical bud of *C*. *verutum* to treat sore throat and epistaxis like in Jajarkot district of Nepal [42,71]. Also this plant is used to treat urinary troubles in our study area which has same application in Karnali and Badagaun of the Gulmi district [63,71]. The practice of using the juice of the root of this plant for typhoid fever and diabetes is unique in our study. In the Mankawanpur district, the whole plant of *D*. *bipinnata* was used to correct thirst, fever, libido, asthma, and jaundice [40]. However, the paste of this plant is useful for tooth aches in our study area, which is justified by one study performed in Badagaun of the Gulmi district [63].

The root of *A. bidentata* contains phytosterols like sitosterol and sigma sterol, which have antibacterial activity, useful for toothaches [87]. The usefulness of this plant for typhoid and tonsillitis is also supported by the antibacterial activity of its ethanolic extract [88]. The plant *A. calva* is used for anthelmintic activity. This activity is justified by the lethality property of this extract against malarial and filarial vector [89]. A study showed that *A. calamus* possesses compounds like papaverine, with a dual inhibitor of the calcium channel and phosphodiesterase in the hexane fraction, and the anticholinergic compound rolipram, which has a phosphodiesterase-4 inhibitor in the ethyl acetate fraction [90]. In addition, one study showed that the anti-asthmatic activity of this plant is devoid of side effects [91]. A decoction of *A*. *sativum* is indigenously taken for gastrointestinal disorders like flatulence, diarrhea, dysentery, and gastritis in our study area. This medicinal property is justified by its reported anti *H. pylori* activity [92,93,94]. During the survey, we encountered the plant *A*. *sessilis*, which is beneficial for bleeding, cuts, and wounds. These properties can be verified by one study, where a chloroform extract of the leaves showed significant wound healing activity [95]. Though the antipyretic activity of this plant is not yet reported, our research findings will open a door for screening for its antipyretic property. The leaves extract of *B*. *purpurea* contain flavonoids and tannins, which exhibit antiulcer activity in rats. This result provides the effectiveness of this plant for the prevention and treatments of gastric ulcers [96]. Additionally, the antimicrobial protein isolated from the seeds of this plant justifies its beneficial effects in gastritis [97]. Folk use of stem and root juice of *B*. *aristata* for typhoid fever, jaundice, and diarrhea is supported by its reported antibacterial activity against *Salmonella typhi* and its hepatoprotective and anti-diarrheal activity, respectively [98,99,100,101,102,103]. One in vivo experiment showed that the same plant contains an excess amount of berberine, an alkaloid responsible for hepatoprotective effects by lowering hepatotoxicity, oxidative and nitrosamine stress, TNF-*α*, and iNOS levels. The inflammatory modulatory effect of this plant in the liver favors its effectiveness in the treatment of jaundice [104]. Experiments performed by Somania et al. [105] showed that an ethanolic extract of *A*. *racemosus* reduced blood glucose level in streptozotocin-induced diabetic rats. Hence, the reported use of this plant in the treatment of diabetes is justified. 

*C*. *asiatica* is indigenously used to cure typhoid fever, cough, tonsillitis, and gastritis in this study area. The purport of indigenous uses of this plant was substantiated by its pharmacological activities (antibacterial, antiulcer, anti-allergic, anti-spasmodic, antitussive, antiviral, and anti-inflammatory activity) shown by compounds like apigenin, luteolin, *α*-pinene, and *β*-pinene [106]. Furthermore, the beneficial effect of *C. asiatica* is justified by its anti-ulcer activity shown in animal models [107]. The reported osteoporotic activity of the ethanolic extract and isolated compound coelogin from *C*. *cristata* justify its traditional use for backache, fractures, and sprains [108]. Its traditional dish, called *Puwa*, is made from the powder of its pseudobulb and is found to be very popular among the villagers for these musculoskeletal disorders. *D*. *carota* is indigenously used to treat jaundice and blindness in this study area. This indigenous use is proved by its antioxidant, hepatoprotective, and intraocular pressure reducing effect [109,110,111,112]. Similarly, *F*. *vulgare* has greater uses for musculoskeletal problems, like fractures and bone weakness. This medicinal potency is also validated by its osteogenesis inducing effect in human mesenchymal cells and its inhibitory effect on osteoclast differentiation and ovariectomy induced bone loss [113,114]. Traditional use of *J*. *curcas* in gingivitis, tonsillitis, and sore throat is proved by its positive in vitro antibacterial property against various susceptible bacteria [115,116]. The usefulness of *J*. *regia* for fungal infection is scientifically confirmed by its fungistatic effect in previous studies [117]. Trigonelline, a potent alkaloid found in *M*. *jalapa*, has a *β* cell protective effect with a good antioxidative potential [118]. This finding supports the clinical use of this plant for diabetes. Additionally, the effectiveness of this plant in the prevention and treatment of diabetes is supported by another hypoglycemic study performed by Zhou et al. [119] in streptozotocin-induced diabetic rats. Experiments performed by Sasa et al. [120] showed that the hypotensive effect of *M*. *charantia* is due to momordin, a saponin present in this plant. This compound has the potential to activate the peroxisome-proliferator activated-receptor (PPAR) delta gene in humans. The activation of this gene is responsible for the formation of high-density lipoproteins (HDL), which leads to a decrease in cardiovascular disorders like atherosclerosis and associated hypertension. 

In our study area, *M*. *alba* is extensively used to reduce toothache. This anti-caries activity is proved by two experiments performed by Lokegaonkar et al. [121] and Islam et al. [122], wherein they found that both the plant extract and the isolated compound 1-deoxynojirimycin worked against *Streptococcus mutans,* a bacterium responsible for causing dental plaque and tooth decay. Similarly, the antibacterial property of this plant against oral pathogens is supported by the isolation of potent antibacterial flavonoids like cyclocommunol (Cy, 1), morusin (Mi, 3), kuwanon G, and kuwanon E (Ku, 4) [123,124]. Hence, the ethnomedicinal use of this plant for toothaches is also pharmacologically validated.

In the Kaski district, *O*. *corniculata* is used to correct gastritis. This beneficial effect is scientifically proved by its gastroprotective effect on experimentally induced gastric ulceration in Wistar rats [125]. *P*. *guajava* has been used to reduce blood pressure in our study area, which is justified by its bioactive compounds like guiajaverin and quercetin having anti-hypertensive property [126]. The ethnomedicinal value of *R*. *ellipticus* juice for cuts and wounds in many parts of our study area is favored by its wound healing effect in animal experiments [127]. We found that a decoction and cooked leaves of *R*. *nepalensis* is used for constipation and its paste is utilized for fungal infections. Literature also showed that the methanolic root extract of this plant has a purgative action in rats and also possesses antifungal activities [125,128]. *S. officinarum* juice contains polyphenolic flavonoids and possesses good antioxidant activity [129]. In addition, this plant is reported to have a hepatoprotective effect, which justifies its ethnomedicinal values for jaundice [130]. Similarly, the ethnomedicinal use of *U*. *parviflora* for jaundice is proved by its hepatoprotective potential in carbon tetrachloride challenged experimental rats [131]. *W*. *fruticosa* contains some antimicrobial terpenes like 2-methoxy-4-(2-propenyl) phenol, 2,6-octadien-1-ol,3,7-dimethyl-(E)-2,6-octadienal, 5-methyl-2-(1-methylethyl) phenol, 3,7 dimethylcyclohexanol, cyclohexanol, and 2-methylene-5-(1-methylethenyl), which may be responsible for the local remedies of this plant for diarrhea and dysentery [132]. An aqueous extract of the *Z*. *armatum* seed was reported to be active against worm infestation caused by *Haemonchus contortus,* which validates its application for intestinal worm in our study area [133]. Locally, flour of *Z*. *mays* is used to reduce blood glucose level. Previous studies proved that phenolic compounds isolated from *Z*. *mays* are effective in reducing complications associated with diabetes mellitus. Hence, the antidiabetic property of this plant in our study area is validated [134]. *Z*. *officinale* contains gingerol as an active ingredient, which possesses cholinergic M_3_ and 5-HT_3_ receptor blocking effects, thereby decreasing gastric emptying time and leading to the prevention of nausea and vomiting [135]. The presence of gingerol, an antiemetic compound, justifies the traditional use of this plant to for the relief of nausea and vomiting.

From the above comparisons of ethnomedicinal uses of medicinal plants with their previous reported uses, it is found that there is a strong variation in their uses within Nepal and other parts of the world. Additionally, the medicinal uses of some plants were newly discovered in our study area and were not encountered in other studies. Such heterogeneity of ethnomedicinal uses of these plants may be due to the predominance in the geographical location and the difference in vegetation. In addition, we found that some of the plants included in our study were already reported for their biological activities and phytochemical constituents. These reported activities and phytochemical constituents are similar to the claimed medicinal uses in this region, which reliability justifies the reported uses. Thus, the ethnomedicinal plant species, which are scientifically validated, are recommended for further studies for advanced pharmacological screening and drug development. 

### 4.6. The Novelty of the Work and Future Prospects

Acharya and Acharya [27] recorded 18 medicinal plants, belonging to 17 families, for treating livestock diseases in Machhapuchchhre (formerly Sardikhola village), in the Kaski district. Similarly, another study, conducted by Bhattarai et al. [28] in Panchase, Kaski, reported 45 plant species belonging to 32 families that treat 34 different ailments. Ethnobotanical study conducted by Adhikari and Fisher [31] reported 54 local plant species from the Ghandruk village located inside ACA. Kunwar [30] also reported 66 plant species from Bhadaure Tamagi of the Kaski district. Among all these reported medicinal plants in this study area, 81 of them were reported in the book “Plants and People of Nepal” [136]. Surprisingly, from this survey we were able to document 83 plant species being used to treat 53 different diseases and ailments for the first time. Among them, 22 medicinal plants appeared to have new ethnomedicinal values. These plants include *A*. *adenophora*, *A*. *bicolor*, *B*. *malabarica*, *C*. *cristata*, *D*. *macrocapnos*, *D*. *racemosus*, *D*. *belophylla*, *E*. *acuminata*, *G*. *hirta*, *H*. *lanceolata*, *M*. *pustulata*, *M*. *chisia*, *M*. *arboreus*, *N*. *cataria*, *P*. *calophylla*, *P*. *peruviana*, *S*. *fasciculata*, *S*. *annuum*, *S*. *pseudocapsicum*, *S*. *torvum*, *T*. *latifolia*, and *U*. *parviflora*. Thus, we can claim that Machhapuchchhre Rural Municipality of the Kaski district is highly rich in medicinal plant resources. This unexplored ethnomedicinal knowledge pool of this area should be well documented, validated, and promoted for the socioeconomic and health benefits of the local people. Phytochemial profile and biological activities of newly encountered plants are yet unknown. This opens the door for researchers to perform phytochemical and pharmacological studies for the discovery of novel bioactive constituents.

## 5. Conclusions

The present study revealed that the Machhapuchchhre Rural Municipality of the Kaski district in Nepal is rich in plant resources that are traditionally used as medicines. The people of this area have been using a variety of plants for treating different diseases and ailments. They have abundant indigenous knowledge about plant collection, dosage form preparation, and their utilization. A total of 105 medicinal plants, belonging to 58 families and 99 genera, which treat 70 different diseases and ailments, were documented. The medicinal values of 22 different plant species were recorded for the first time in the district. The medicinal properties of the plants were justified by comparing them with relevant literature published from different parts of the world. The positive correlation between the traditional uses of the reported plants and their experimentally proven pharmacological activities was established. 

To recapitulate, this study provides authentic data regarding the ethnomedicinal uses of the local flora of this rural region and will draw the attention of pharmacognosists, pharmacologists, phytochemists, and traditional healers for conducting further research to find therapeutically active natural products. We recommend that the claimed medicinal plants of this area should be protected using appropriate conservation measures and that a scientific assessment of the plant-lore in the district is urgently needed.

## Figures and Tables

**Table 1:** Enumeration of medicinal plant species used with their parts used, preparation form, local names, family names, mode of application and ailments treated.

S.N.	Botanical Name Voucher Number	Family	Local Name	Habit	Parts Used	Preparation	Mode of Application	Ailments Treated/ Uses	Previous Uses Reported References
1.	*Abelmoschus manihot* var. *pungens* Hochr. NAH-25	Malvaceae	Kapasi	Herb	Root	Decoction and juice	Oral	Fever	[72]
2.	*Achyranthes bidentata* Bl. NAH-75	Amaranthaceae	Datiwan	Herb	Root	Juice	Oral	Typhoid, Tonsillitis, Urine retention	[7,36,39,40,59]
					Stem	Powder	Chewing	Toothache	
3.	*Acmella calva* (DC.) R.K. Jansen NAH-50	Asteraceae	Marathi	Herb	Fruit	Pickle	Oral	Intestinal worm, Gastritis, Flatulence	[69]
4.	*Acorus calamus* L. NAH-35	Acoraceae	Bojho	Herb	Rhizome	Chewable (rhizome)	Oral	Cough, Chest pain, Asthma	[13,22,31,36,40,44,46,47,49,52,53,57,58,63,69,71,73]
5.	*Ageratina adenophora* (Spreng.) R. M. King & H. Rob. NAH-52	Asteraceae	Banmara	Shrub	Leaf	Juice	Topical	Bleeding, Cuts and Wounds	None
6.	*Aleuritopteris bicolor* (Roxb.) Fraser-Jenk. NAH-55	Pteridaceae	Dankernu	Fern	Whole plant	Juice	Oral	Diarrhea, Dysentery, Gastritis	None
7.	*Allium sativum* L. NAH-112	Amaryllidaceae	Lasun	Herb	Bulb	Decoction	Oral	Gastritis, Flatulence, Diarrhea, Dysentery	[6,7,59,65,70]
					Bulb	Paste	Topical	Snakebite	
8.	*Aloe vera* (L.) Burm.f. NAH-60	Xanthorrhoeaceae	Gheukumari	Herb	Leaf	Gel	Topical	Burns, Boils	[28,36,40,44,66,70]
9.	*Alternanthera sessilis* (L.) R. Br. Ex DC. NAH-133	Amaranthaceae	Bhiringi Jhar	Herb	Leaf	Juice	Topical	Bleeding	[11,44,52,69]
					Leaf	Juice and paste	Topical	Cuts and Wounds	
10.	*Anaphalis triplinervis* (Sims) Sims ex C.B. Clarke NAH-120	Asteraceae	Buki ful	Herb	Whole plant	Juice	Oral	Post-partum hemorrhage	[41,60,74,75]
11.	*Artemisia dubia* L. ex B. D. Jacks. NAH-110	Asteraceae	Tite pati	Herb	Leaf	Juice and paste	Topical	Scabies	[11,36,46,50,57,74,76]
12.	*Asparagus racemosus* Willd. NAH-109	Asperagaceae	Kurilo/ Satawari	Shrub	Whole plant	Juice and decoction	Oral	Hypertension, Diabetes mellitus	[13,27,30,31,36,39,40,41,44,47,48,49,53,57,59,63,71,77,78]
13.	*Azadirachta indica* A. Juss NAH-121	Meliaceae	Neem	Tree	Bark	Juice	Oral	Fever, Skin disorders	[11,28,36,38,39,40,44,49,52,53,76,79]
14.	*Bauhinia malabarica* Roxb. NAH-67	Fabaceae	Tanki	Tree	Bark and root	Juice and decoction	Oral	Typhoid, tonsillitis	None
15.	*Bauhinia purpurea* L. NAH-01	Fabaceae	Koiralo	Tree	Bark	Decoction	Oral	Gastritis	[36,69]
16.	*Berberis aristata* DC. NAH-124	Berberidaceae	Chutro	Shrub	Bark and root	Juice	Oral	Typhoid, Fever, Diarrhea, Jaundice	[6,13,22,30,31,36,40,41,42,46,50,51,52,57,60,63,69,71,73,74,75,77,80]
17.	*Bergenia ciliata* (Haw.) Sternb. NAH-36	Saxifragaceae	Pakhanbed	Herb	Rhizome	Paste	Topical	Sprain, Fracture	[7,13,22,36,37,40,42,50,51,52,56,57,59,60,63,66,70,73,74,75,76,77,78,79,80]
					Rhizome	Powder along with rice flour in ghee	Oral	Body ache, Backache	
18.	*Betula alnoides* Buch.-Ham.ex D. Don NAH-22	Betulaceae	Saur	Tree	Bark	Juice	Oral	Fever, Backache	[7,63,73,78]
19.	*Bryophyllum pinnatum* (Lam.) Oken NAH-14	Crassulaceae	Ajambari	Herb	Flower	Juice	Aural	Earache	[69,79]
20.	*Centella asiatica* (L.) Urb. NAH-104	Apiaceae	Ghod tapre	Herb	Whole plant	Paste and juice	Oral	Typhoid, Cough, Tonsillitis, Gastritis	[11,27,30,31,36,39,41,42,44,46,47,49,53,57,58,59,63,69,71,79]
21.	*Chenopodium album* L. NAH-12	Amaranthaceae	Bethe	Herb	Leaf and fruit	Juice and decoction	Oral	Gastritis, Flatulence, Stomachache	[22,36,38,53,57,65]
22.	*Cinnamomum tamala* (Buch.-Ham.) T. Nees & Eberm NAH-107	Lauraceae	Tejpat/ Dalchini	Tree	Leaf	Decoction	Oral	Fever, Stomachache, Gastritis	[31,36,39,40,47,50,57,58,63,73,80]
23.	*Cirsium verutum* (D. Don) Spreng. NAH-70	Asteraceae	Thakailo/Thakali kanda	Herb	Bud and root	Chewable (bud and root)	Oral	Sore throat, Nose bleeding	[30,42,45,57,63,76]
					Root	Juice	Oral	Typhoid, Diabetes mellitus	
24.	*Cissampelos pareira* L. NAH-71	Menispermacae	Thulo Batul pate	Climber	Whole plant	Decoction	Oral	Postpartum hemorrhage	[11,28,36,41,44,47,52,57,59,69,74,80]
25.	*Citrus medica* L. NAH-11	Rutaceae	Bimiro	Tree	Root and fruit	Juice	Oral	Intestinal worms	[80]
26.	*Citrus aurantiifolia* (Christm.) Swingle NAH-93	Rutaceae	Kagati	Tree	Fruit	Juice, juice with honey	Oral	Thirst, Jaundice, Anorexia, Pimple, Skin disorders	[7,39,58,59]
27.	*Citrus limon* (L.) Osbeck NAH-59	Rutaceae	Nibuwa	Tree	Fruit	Juice	Oral	Food poisoning, Blood purification	[11,39,48,53,65,66,76]
28.	*Coelogyne cristata* Lindl. NAH-122	Orchidaceae	Kadam	Orchid	Pseudobulb	Powder mixed with cooked rice flour in butter (Puwa)	Oral	Backache, Fracture, Sprain	None
29.	*Coffea Arabica* L. NAH-63	Rubiaceae	Kafi	Shrub	Fruit	Decoction	Oral	Lethargy, Headache	[64]
30.	*Colocasia esculenta* (L.) Schott NAH-20	Araceae	Karkalo	Herb	Stem	Latex	Topical	Snakebite	[30,40,53]
					Whole plant and root	Decoction and vegetable curry	Oral	Constipation	
31.	*Cucurbita pepo* L. NAH-26	Cucurbitaceae	Farsi	Herb	Fruit	Boiledfruit	Oral	Jaundice, Gastritis	[22,64,66]
32.	*Curcuma longa* L. NAH-111	Zingiberaceae	Besar/Haledo	Herb	Rhizome	Decoction	Oral	Fever, Sore throat, Sinusitis, Common cold	[31,38,52,53,58,59,75,80]
33.	*Cuscuta reflexa* Roxb. NAH-92	Convolvulaceae	Aakase beli	Herb	Whole plant	Juice	Oral	Jaundice	[11,22,31,39,40,41,43,44,47,49,51,52,53,59,63]
34.	*Cyathea spinulosa* Wall. ex Hook. NAH-32	Cyatheaceae	Chattre	Fern	Soft pith	Decoction prepared in ghee	Oral	Fracture, Body ache	[30]
35.	*Cynodon dactylon* (L.) Pers. NAH-74	Poaceae	Dubo	Grass	Whole plant	Juice	Oral	Diarrhea, Dysentery, Intestinal worm, Flatulence	[6,11,39,44,45,49,51,53,57,58,63,70,73,80]
36.	*Dactylicapnos macrocapnos* (Prain) Hutch. NAH-16	Papaveraceae	Bichkane Jhar	Climber	Whole plant	Squeezed Juice	Topical	Corneal scar	None
37.	*Daucus carota* L. NAH-72	Apiaceae	Gajar	Herb	Rhizome	Chewable (rhizome)	Oral	Jaundice, Blindness	[39,56,65]
38.	*Desmostachya bipinnata* (L.) Stapf. NAH-04	Poaceae	Kush	Grass	Root	Paste	Topical	Toothache	[40,63]
39.	*Dinetus racemosus* (Roxb.) Buch.-Ham. ex Sweet NAH-62	Convolvulaceae	Badimal	Climber	Rhizome	Decoction	Oral	Post-partum hemorrhage	None
40.	*Dioscorea belophylla* (Prain) Voight ex Haines NAH-07	Dioscoreaceae	Ban Tarul	Climber	Tuber	Boiled tuber	Oral	Constipation	None
41.	*Drepanostachyum falcatum* (Nees) Keng f. NAH-34	Poaceae	Nigalo	Grass	Stem	Dust outside the stem	Topical	Tinea pedis	[7,30]
42.	*Drymaria diandra* Blume NAH-138	Caryophyllaceae	Abijalo	Herb	Whole plant	Paste and juice	Oral	Gastritis, Flatulence, Nausea, Vomiting	[28,39,51,52,53,58,63,78,80]
						Stem vapor	Inhalation	Sinusitis	
43.	*Eclipta prostrata* (L.) L. NAH-61	Asteraceae	Bhiringe	Herb	Leaf	Juice	Topical	Bleeding, Wound	[11,27,47,52,53,69,78]
44.	*Eleusine coracana* (L.) Gaertn. NAH-30	Poaceae	Kodo	Grass	Fruit	Cooked flour	Oral	Cough	[69]
45.	*Emilia sonchifolia* (L.) DC. ex DC. NAH-10	Asteraceae	Salha ko jhar	Herb	Whole plant	Juice	Topical	Bleeding, Cuts and Wounds	[11]
46.	*Engelhardia spicata* Lesch. ex Blume NAH-77	Juglandaceae	Mauwa	Tree	Bud	Paste	Topical	Tinea pedis	[28,39,58,80]
47.	*Erythrina stricta* Roxb. NAH-23	Fabaceae	Fadelo	Tree	Bark	Juice and decoction	Oral	Typhoid, Sore throat	[78]
48.	*Eurya acuminata* DC. NAH-118	Pentaphylacaceae	Jhyanu	Shrub	Bud	Juice	Oral	Typhoid, Tonsillitis, Sore throat	None
49.	*Ficus religiosa* L. NAH-56	Moraceae	Pipal	Tree	Leaf	Juice	Topical	Cuts and Wounds	[11,36,38,40,49,57,63,66,69]
50.	*Foeniculum vulgare* Mill. NAH-99	Apiaceae	Sonp	Herb	Fruit	Powder mixed with cooked rice flour in butter (Puwa)	Oral	Bone weakness, Fracture	[56,64,81]
51.	*Gonostegia hirta* (Blume ex Hassk.) Miq. NAH-08	Urticaceae	Aaternu	Herb	Leaf	Paste	Topical	Boils	None
52.	*Hoya lanceolata* Wall. ex D. Don NAH-09	Asclepiadaceae	Thirjo	Shrub	Whole plant	Juice	Oral	Body ache	None
53.	*Imperata cylindrica* (L.) Raeusch. NAH-45	Poaceae	Siru	Grass	Root	Juice	Oral	Typhoid	[22,38,57,63]
54.	*Jatropha curcas* L. NAH-38	Euphorbiaceae	Sajiwan	Tree	Leaf and stem	Juice and paste	Gargling	Gingivitis, Tonsillitis, Sore throat	[11,36,44,47,57,63]
						Latex	Topical	Fungal infection	
55.	*Juglans regia* L. NAH-27	Juglandaceae	Okhar	Tree	Peels of fruit	Paste	Topical	Tinea pedis, Fungal infection	[13,31,37,46,47,58,60,63,71,74,75,77]
56.	*Linum usitatissimum* L. NAH-88	Linaceae	Aatasi	Herb	Seed	Roast	Oral	Lethargy, Intestinal worms	[53]
57.	*Litsea cubeba* (Lour.) Pers. NAH-40	Lauraceae	Siltimur	Tree	Seed	Decoction and spice	Oral	Gastritis, Flatulence, Indigestion, Gastric troubles	[31,40]
					Bark	Paste	Topical	Cuts and Wounds, Fracture	
58.	*Lycopersicon esculentum* Mill. NAH-57	Solanaceae	Golveda	Herb	Fruit	Juice	Oral	Burns, Boils	[39,65,80]
59.	*Lyonia ovalifolia* (Wall.) Drude NAH-94	Ericaceae	Thaune	Tree	Bud	Paste	Topical	Scabies	[36,39,41,58,59,63]
60.	*Macaranga pustulata* King ex Hook.f. NAH-19	Euphorbiaceae	Mallato	Tree	Stem	Latex	Topical	Boils	None
61.	*Maesa chisia* Buch. Ham. ex. D. Don NAH-02	Primulaceae	Bilaune	Shrub	Fruit and Root	Juice and paste	Topical	Scabies	None
62.	*Mahonia napaulensis* DC. NAH-142	Berberidaceae	Bhutro/ Jamane mandro	Shrub	Stem	Latex	Topical	Conjunctivitis	[30,40,51,59,63]
63.	*Malvaviscus arboreus* Cav. NAH-47	Malvaceae	Barhamase ful/khursani ful	Shrub	Root	Juice and decoction	Oral	Fever	None
64.	*Mirabilis jalapa* L. NAH-49	Nyctaginaceae	Seto malati	Shrub	Root	Paste	Topical	Inflammation	[69]
					Rhizome	Chewable, Juice and decoction	Oral	Diabetes mellitus, Gastritis, Flatulence, Body ache	
65.	*Mentha arvensis* L. NAH-85	Lamiaceae	Babari	Herb	Flower	Juice and decoction	Oral	Thirst	[36,49,63,80]
66.	*Mentha spicata* L. NAH-86	Lamiaceae	Pudina	Herb	Whole plant	Juice, Paste and pickle	Oral	Diarrhea, Dysentery, Urine retention, Stomachache, indigestion	[7,11,22,30,44,47,53,57,59,63,73,81]
67.	*Momordica charantia* L. NAH-28	Cucurbitaceae	Tite karela	Herb	Fruit	Juice, decoction, and boiled fruit	Oral	Hypertension	[11,52,66]
68.	*Morus alba* L. NAH-119	Moraceae	Kiu kafal	Tree	Bark	Juice and paste	Topical	Toothache	[66,76,81]
69.	*Musa* x *paradisiaca* L. NAH-06	Musaceae	Kera	Stoloniferous plant	Corm	Juice and decoction	Oral	Thirst, Fever	[11,31,53,80]
70.	*Myrica esculenta* Buch.-Ham. ex D. Don NAH-18	Myricaceae	Ban Kafal	Tree	Bark	Chewable (bark)	Oral	Diabetes mellitus, Toothache	[13,28,51,58,78,80]
71.	*Nepeta cataria* L. NAH-83	Lamiaceae	Charpate	Herb	Leaf, whole plant	Juice	Topical	Cuts and Wounds, Bleeding	None
72.	*Nephrolepsis cordifolia* (L.) C. Presl NAH-39	Nephrolepidaceae	Pani amala	Fern	Tuber	Juice	Oral	Jaundice, Diabetes mellitus, Hematuria	[11,12,30,36,78]
73.	*Nicotiana tobacum* L. NAH-80	Solanaceae	Surti/Kacho paat	Herb	Leaf	Paste and expressed juice	Topical	Infected wounds, Pediculosis	[49,71,78]
74.	*Ocimum tenuiflorum* L. NAH-102	Lamiaceae	Tulsi	Shrub	Leaf and whole plant	Decoction and juice	Oral	Heart failure, Flu, cardiac stimulant	[49,52,53,63,78]
75.	*Oryza sativa* L. NAH-48	Poaceae	Dhan	Grass	Fruit	Decoction	Oral	Inflammation	[7]
						Roasted fruit	Oral	Cough	
76.	*Oxalis corniculata* L. NAH-58	Oxalidaceae	Chari amilo	Herb	Whole plant	Decoction and juice	Oral	Flatulence, Gastritis, Diarrhea, Dysentery, Fever, Common cold, Typhoid	[11,36,44,45,48,49,57,58,59,63,64,71,80]
77.	*Periploca calophylla* (Wight) Falc. NAH-31	Apocynaceae	Chautajor	Shrub	Leaf and stem	Powder mixed with cooked rice flour in butter (Puwa)	Oral	Body ache, Backache	None
78.	*Physalis peruviana* L. NAH-13	Solanaceae	Isamgol	Herb	Fruit	Decoction	Oral	Fever, Thirst	None
79.	*Pogostemon benghalensis* (Brum.f.) Kuntze NAH-134	Lamiaceae	Rudilo	Shrub	Leaf	Juice	Oral	Sore throat, Typhoid	[11,40,49,63,78]
80.	*Potentilla lineata* Trev. NAH-69	Rosaceae	Bajradanti	Herb	Leaf and root	Juice	Topical	Toothache	[58]
81.	*Prunus persica* (L.) Batsch NAH-100	Rosaceae	Aaru	Tree	Bark and leaf	Decoction	Oral	Typhoid, Sore throat	[11,49,58,65,66,70]
82.	*Psidium guajava* L. NAH-43	Myrtaceae	Belauti/Aamba	Tree	Bud and bark	Juice	Oral	Diarrhea, Dysentery, Hypertension	[7,11,31,39,45,49,58,59,63,66,78,80]
83.	*Rhododendron arboreum* Sm. NAH-108	Ericaceae	Laali Gurans	Tree	Flower	Chewable(flower petal)	Oral	Throat obstruction	[22,30,31,36,37,39,40,42,47,51,59,63,65,73,74,78,80]
						Juice	Oral	Diarrhea, Dysentery, Dry cough due to allergy	
84.	*Rubus ellipticus* Sm. NAH-117	Rosaceae	Ainselu	Shrub	Bud and roots	Juice	Oral	Diabetes mellitus	[22,28,30,31,36,39,40,45,51,58,59,63,80]
					Buds and leaf	Juice	Topical	Cutsand Wounds	
85.	*Rumex nepalensis* Spreng. NAH-115	Polygonacae	Halhale	Herb	Whole plant	Juice and paste	Topical	Dislocated bone, Fracture, Sprain, Eye troubles	[22,30,36,41,42,45,46,51,53,56,58,60,63,64,71,74,75,80]
					Leaf	Decoction and cooked leaves	Oral	Constipation	
					Leaf	Paste	Topical	Fungal infection, Skin disorders	
					Root	Juice	Oral	Diarrhea	
86.	*Saccharum officinarum* L. NAH-29	Poaceae	Ukhu	Grass	Stem	Juice	Oral	Jaundice	[7]
87.	*Saurauia fasciculata* Wall. NAH-42	Actinidiaceae	Goban	Tree	Bark	Juice	Oral	Thirst, Postpartum hemorrhage, Fever	None
88.	*Schima wallichii* Choisy NAH-78	Theaceae	Chilaune	Tree	Bark	Latex	Topical	Bleeding, Cuts and Wounds	[31,36,49,63,78,80]
89.	*Solanum annuum* C.V. Morton NAH-51	Solanaceae	Khursani	Herb	Fruit	Fried in oil	Topical	Snakebite, Scabies	None
90.	*Solanum melongena* L. NAH-46	Solanaceae	Bhanta	Herb	Unripe fruit	Decoction	Oral	Fever	[7]
					Bud	Paste	Topical	Burns, Boils	
91.	*Solanum pseudocapsicum* L. NAH-03	Solanaceae	Tite bee	Shrub	Fruit	Juice	Topical	Headache, Toothache	None
92.	*Solanum torvum* Sw. NAH-95	Solanaceae	Kantakari (Thulo Bihi)	Shrub	Fruit	Smoke	Inhalation	Toothache	None
93.	*Stephania japonica* (Thunb.) Miers NAH-82	Menispermacae	Chillo badulpate	Climber	Leaf	Decoction	Oral	Postpartum hemorrhage	[31]
94.	*Swertia chirayita* L. NAH-113	Gentianaceae	Chiraaito	Herb	Leaf	Juice and decoction	Oral	Fever, Headache, Common cold, Gastritis, Constipation	[22,28,39,40,41,47,50,51,57,63,66,78]
95.	*Tectaria coadunata* (J. Sm.) C. Chr. NAH-41	Dryopteridaceae	Kalo kuthurke	Fern	Root	Juice	Oral	Diarrhea, Dysentery	[28]
96.	*Thysanolaena latifolia* (Roxb. ex Hornem) Honda NAH-21	Poaceae	Amriso	Grass	Root	Paste	Topical	Breast engorgement	None
						Decoction	Oral	Fever	
97.	*Trigonella foenum-graceum* L. NAH-143	Fabaceae	Methi	Herb	Fruit	Soup of roasted fruit	Oral	Common cold, Cough	[22,39,52,56]
98.	*Urtica parviflora* Roxb. NAH-79	Urticaceae	Sisno	Shrub	Leaf and Bud	Juice and decoction	Oral	Blindness, Jaundice, Urine retention	None
99.	*Viburnum mullaha* Buch.-Ham. ex D. Don NAH-89	Adoxaceae	Molo	Tree	Fruit	Juice	Oral	Poisoning	[30]
100.	*Viola canescens* Wall. NAH-24	Violaceae	Aankhle jhar	Herb	Leaf	Paste	Topical	Gout, Joint pain	[58,70]
101.	*Woodfordia fruticosa* (L.) Kurz NAH-17	Lythraceae	Dhairo	Shrub	Flower	Juice	Oral	Diarrhea, Dysentery	[11,44,47,49,57,59,63,70,78,79]
102.	*Zanthoxylum armatum* DC. NAH-144	Rutaceae	Aakhe Timur	Shrub	Fruit	Juice and paste	Topical	Snakebite, Scabies	[13,30,31,36,37,40,42,46,47,50,51,57,59,60,63,70,71,73,76,78,80]
						Juice and decoction	Oral	Gastritis, Intestinal worms, Toothache, Poisoning	
103.	*Zea mays* L. NAH-15	Poaceae	Makai	Grass	Seed	Flour	Oral	Diabetes mellitus	[6,7,56,65,66]
104.	*Zingiber officinale* Roscoe NAH-64	Zingiberaceae	Aduwa	Herb	Rhizome	Paste	Topical	Snakebite	[6,11,22,49,50,52,58,73]
						Decoction	Oral	Vomiting, Stomachache, Common cold	
105.	*Ziziphus mauritiana* Lam. NAH-44	Rhamnaceae	Bayer	Tree	Seed	Powder	Chewing	Chickenpox, Measles	[6,11,30,47,49,53,57,63,65,66]

**Table 2:** List of plant species used for specific categories of diseases and ailments.

Disease and Ailment Categories	Name of Plants	No of Plants
Musculoskeletal	*B*. *ciliata*, *B*. *alnoides*, *C*. *cristata*, *C*. *spinulosa*, *F*. *vulgare*, *H*. *lanceolata*, *L*. *cubeba, M*. *jalapa*, *O*. *sativa, P*. *calophylla*, *R*. *nepalensis*, *V*. *canescens*	12
Digestive	*A*. *bidentata*, *A*. *calva*, *A*. *bicolor*, *A*. *sativum*, *B*. *purpurea*, *B*. *aristata*, *C*. *asiatica*, *C*. *album*, *C*. *tamala*, *C*. *medica*, *C*. *aurantifolia*, *C*. *esculenta*, *C*. *pepo*, *C*. *dactylon*, *D*. *carota*, *D*. *bipinnata*, *D*. *bulbophylla*, *D*. *diandra*, *J*. *curcas*, *L*. *usitatissimum*, *L*. *cubeba*, *M*. *jalapa*, *M*. *spicata*, *N*. *cordifolia*, *O*. *corniculata*, *P*. *guajava*, *R*. *arboreum*, *R*. *nepalensis*, *S*. *officinarum*, *S*. *pseudocapsicum*, *S*. *torvum*, *S*. *chirayita*, *T*. *coadunata*, *U*. *parviflora*, *W*. *fruticosa*, *Z*. *armatum, Z*. *officinale*	37
Eye	*D*. *carota*, *M*. *napaulensis*, *R*. *nepalensis*, *U*. *parviflora*	4
Ear	*B*. *pinnatum*, *M*. *arboreus*	2
Circulatory	*A*. *adenophora*, *A*. *sessilis*, *A*. *racemosus*, *C*. *verutum*, *C*. *limon*, *E*. *prostrata*, *E*. *sonchifolia*, *M*. *charantia*, *N*. *cataria*, *O*. *tenuiflorum, P guagava*	11
Neurological	*C*. *arabica*, *S*. *pseudocapsicum*, *S*. *chirayita*	3
Respiratory	*A*. *bidentata*, *A*. *calamus*, *B*. *malabarica*, *C*. *asiatica*, *C*. *verutum*, *C*. *longa*, *D*. *diandra*, *E*. *coracana*, *E*. *stricta*, *E. acuminata*, *J. curcas*, *O. sativa*, *O*. *corniculata, P*. *benghalensis, P*. *persica, R*. *arboreum*, *S*. *chirayita, T*. *foenum*-*graceum, Z*. *officinale*	19
Urinary System	*A*. *bidentata, M*. *spicata*, *N*. *cordifolia*, *U*. *parviflora*	4
Skin	*A*. *adenophora, A*. *vera*, *A*. *sessilis, A*. *dubia*, *A*. *indica*, *C*. *aurantifolia*, *D*. *falcatum*, *E*. *prostrata, E*. *sonchifolia*, *E*. *spicata*, *F*. *religiosa, G*. *hirta*, *J. curcas*, *J*. *regia*, *L*. *cubeba*, *L*. *esculentum*, *L*. *ovalifolia*, *M*. *pustulata*, *M*. *chisia*, *N*. *cataria*, *N*. *tobacum*, *R*. *ellipticus*, *R*. *nepalensis*, *S*. *wallichii*, *S*. *annuum*, *S*. *melongena*, *Z*. *armatum*	27
Endocrine, Metabolic and Nutritional	*A*. *racemosus, C*. *verutum, C*. *aurantifolia*, *M*. *jalapa*, *M*. *esculenta*, *N*. *cordifolia*, *P. peruviana*, *R. ellipticus*, *V. canescens*, *Z. mays*	10
Pregnancy, Childbearing, Family Planning	*A*. *triplinervis*, *C*. *pareira*, *D*. *racemosus*, *S*. *fasciculata, S*. *japonica*	5
Female Genital System (including Breast)	*T*. *latifola*	1
General and Unspecified	*A*. *manihot*, *A*. *bidentata, A*. *calamus*, *A*. *sativum*, *A*. *indica*, *B*. *malabarica*, *B*. *aristata*, *B*. *alnoides*, *C*. *asiatica*, *C*. *tamala*, *C*. *verutum*, *C*. *limon*, *C*. *arabica*, *C*. *esculenta*, *C*. *longa*, *E*. *stricta*, *E*. *acuminata*, *I*. *cylindrica*, *L*. *usitatissimum*, *M*. *arboreus*, *M*. x *paradisiacal, O*. *tenuiflorum*, *O*. *corniculata*, *P*. *peruviana*, *P*. *benghalensis*, *P*. *persica*, *S*. *fasciculata*, *S*. *annuum*, *S*. *melongena*, *S*. *chirayita*, *T. latifola*, *V. mullaha*, *Z. armatum*, *Z. officinale*, *Z. mauritiana*	35

**Table 3:** Categories of diseases and ailments based on body system, along with their biomedical and emic use reports.

Ailments Categories	Biomedical Terms	Local Terms (Emic Use Reports)
General and Unspecified	Chest pain	*Chati dukheko*
	Chickenpox	*Theula*
	Fever	*Jworo aayeko*
	Flu	*Bela Bela dekhaparne rughakoki, joro*
	Food poisoning	*Khana ma bish pareko*
	Lethargy	*Aalasya vayeko, alchi lageko*
	Measles	*Dadura*
	Snakebite	*Sarpa le tokeko*
	Typhoid	*Kukhat pareko*
Digestive	Anorexia	*Khana ruchi nahune/ bhok nalagne/ aman hune*
	Constipation	*Kabjiyet hune/ pet safa nahune/ disha garda garo hune*
	Diarrhea	*Pakhala lageko/ cherpate chaleko/ pani jasto patalo disha aaune*
	Dysentery	*Aaun pareko*
	Emesis	*Banta garaune*
	Flatulence	*Pet ma hawa variyeko/ bayu gola le pet dukhne*
	Gastric troubles	*Pet ko gadbadi/ pet dhadiyeko*
	Gastritis	*Gastric vayeko/ mukh ma amilo pani aaune/ chati polne*
	Gingivitis	*Gija sunniyeko*
	Indigestion	*Apach/ khana apach hune*
	Intestinal worms infestation	*Pet ma juka pareko*
	Jaundice	*Pahele rog/ jaundice vayeko*
	Nausea	*Banta hola jasto hune/wakwak lagne*
	Stomachache	*Pet dukheko*
	Throat obstruction	*Ghanti ma adkeko, ghanti ma kei kura aljeko*
	Toothache	*Dant dukheko/ dant kirale khayeko*
	Vomiting	*Banta hune/ ulti hune*
Eye	Blindness	*Aandho hune*
	Conjunctivitis	*Aankha pakne rog*
	Corneal scar	*Aankha ma phulo parne*
	Eye troubles	*Aankha ka samasyaharu*
Ear	Earache	*Kan dukheko*
Circulatory	Bleeding	*Dherai ragat bagne*
	Blood purification	*Ragat safa garne*
	Heart failure	*Mutu fail hune/ hridayaghat*
	Hypertension	*Uchha raktachap, blood pressure badeko*
Musculoskeletal	Backache	*Dhad dhukeko/ kammar dukheko*
	Bodyache	*Jyan dukheko*
	Bone weakness	*Haddi ko kamjori*
	Dislocated bone	*Haddi bhachiyera thau sareko*
	Fracture	*Haddi futeko/bhachiyeko*
	Inflammation	*Sunniyeko*
	Joint pain	*Jorni ko dukhai*
	Sprain	*Markeko*
Neurological	Headache	*Tauko dukheko*
Respiratory	Asthma	*Dam vayeko/ sas ferna garo hune rog*
	Common cold	*Rugha khoki lagne*
	Cough	*Khoki lageko/ kaso lageko*
	Dry cough due to allergy	*Allergy le garda hune sukka khoki*
	Nose bleeding	*Naak bata ragat bagne*
	Sinusitis	*Pinas bhayeko*
	Sore throat	*Ghanti basne*
	Tonsillitis	*Ghanti dukheko*
Skin	Boils	*Pilo*
	Burns	*Poleko/ dadeko*
	Cuts	*Kateko*
	Fungal infections	*Sarir ma daj aaune*
	Infected wounds	*Ghau pakeko*
	Pediculosis	*Sarirma jumra parne*
	Pimple	*Dandifor aayeko*
	Scabies	*Luto/ suke luto aayeko*
	Skin disorders	*Chala ko samasya/ chala ko rog/ charma rog*
	Tinea pedis/ ring worm	*Kash ma daj aayeko*
	Wounds	*Ghau lageko*
Endocrine, Metabolic and Nutritional	Diabetes mellitus	*Chini rog, Madhumeha*
	Gout	*Bath rog*
	Thirst	*Kharo hune*
Urinary System	Hematuria	*Pisab ma ragat dekhine*
	Urine retention	*Pisab banda vayeko*
Pregnancy, Childbearing, Family Planning	Post-partum hemorrhage	*Sutkeri veyepachi dherai ragat bagne/ khanaro hune*
Female Genital System (including Breast)	Breast engorgement	*Stan ma thunelo aayeko*
